# Impact of cattle on the abundance of indoor and outdoor resting malaria vectors in southern Malawi

**DOI:** 10.1186/s12936-021-03885-x

**Published:** 2021-08-26

**Authors:** Monicah M. Mburu, Kennedy Zembere, Themba Mzilahowa, Anja D. Terlouw, Tumaini Malenga, Henk van den Berg, Willem Takken, Robert S. McCann

**Affiliations:** 1 School of Public Health and Family Medicine, College of Medicine, University of Malawi, Blantyre, Malawi; 2 Laboratory of Entomology, Wageningen University and Research, Wageningen, The Netherlands; 3 Macha Research Trust, Choma, Zambia; 4 Malawi-Liverpool-Wellcome Trust, Blantyre, Malawi; 5 MAC Communicable Diseases Action Centre, Blantyre, Malawi; 6 Liverpool School of Tropical Medicine, Liverpool, UK; 7 Center for Vaccine Development and Global Health, University of Maryland School of Medicine, Baltimore, USA

**Keywords:** Anophelines, Blood-meal hosts, Cattle, Indoors, Outdoors, Resting, Zooprophylaxis

## Abstract

**Background:**

Understanding the blood feeding preferences and resting habits of malaria vectors is important for assessing and designing effective malaria vector control tools. The presence of livestock, such as cattle, which are used as blood meal hosts by some malaria vectors, may impact malaria parasite transmission dynamics. The presence of livestock may provide sufficient blood meals for the vectors, thereby reducing the frequency of vectors biting humans. Alternatively, the presence of cattle may enhance the availability of blood meals such that infectious mosquitoes may survive longer, thereby increasing the risk of malaria transmission. This study assessed the effect of household-level cattle presence and distribution on the abundance of indoor and outdoor resting malaria vectors.

**Methods:**

Houses with and without cattle were selected in Chikwawa district, southern Malawi for sampling resting malaria vectors. Prokopack aspirators and clay pots were used for indoor and outdoor sampling, respectively. Each house was sampled over two consecutive days. For houses with cattle nearby, the number of cattle and the distances from the house to where the cattle were corralled the previous night were recorded. All data were analysed using generalized linear models fitted with Poisson distribution.

**Results:**

The malaria vectors caught resting indoors were *Anopheles gambiae sensu* *stricto* (*s.s*.), *Anopheles arabiensis* and *Anopheles funestus* *s.s*. Outdoor collections consisted primarily of *An. arabiensis*. The catch sizes of indoor resting *An. gambiae sensu* *lato* (*s.l*.) were not different in houses with and without cattle (P = 0.34). The presence of cattle near a house was associated with a reduction in the abundance of indoor resting *An. funestus* *s.l.* (P = 0.04). This effect was strongest when cattle were kept overnight ≤ 15 m away from the houses (P = 0.03). The blood meal hosts varied across the species.

**Conclusion:**

These results highlight differences between malaria vector species and their interactions with potential blood meal hosts, which may have implications for malaria risk. Whereas *An. arabiensis* remained unaffected, the reduction of *An. funestus* *s.s*. in houses near cattle suggests a potential protective effect of cattle. However, the low abundance of mosquitoes reduced the power of some analyses and limited the generalizability of the results to other settings. Therefore, further studies incorporating the vectors’ host-seeking behaviour/human biting rates are recommended to fully support the primary finding.

**Supplementary Information:**

The online version contains supplementary material available at 10.1186/s12936-021-03885-x.

## Background

An estimated 229 million cases of malaria occurred globally in 2019, with 94% of the cases in Africa [1]. Rearing of livestock such as cattle is an important part of people’s livelihoods in rural areas of Africa [2], where malaria risk is higher than in urban areas [3]. At the household-level, the presence of cattle may reduce [4] or enhance [5, 6] the risk of malaria infection. Although clear differences in host preference exist among malaria vector species [7, 8], the final blood meal host of a mosquito depends on a complex set of factors, such as the availability and abundance of hosts [8]. For instance, of the dominant malaria vector species in Africa, *Anopheles gambiae sensu* *stricto* (*s.s*.), *Anopheles coluzzii* and *Anopheles funestus* *s.s*. are highly anthropophagic, while *Anopheles arabiensis* is more variable in its feeding behaviour, readily feeding on cattle in addition to humans [9–12]. Following the use of long-lasting insecticidal nets (LLINs), humans may become inaccessible for a blood meal by malaria vectors. As a result, the vectors may prefer to feed on the next available non-human hosts [8] such as cattle [13]. Conflicting results have been reported on studies assessing the effect of cattle presence in relation to the risk of malaria infection. For instance, in Tanzania, while the human blood index (HBI) of *An. arabiensis* and *An. funestus sensu* *lato* (*s.l*.) was lower in households with cattle than those without cattle, that of *An. gambiae* *s.s*. was not different [4]. Similar results were observed in the Gambia for *An. arabiensis* and *An. gambiae* *s.s*. [14]. In relation to sporozoite rates, Mayagaya et al*.* [4] found that the infection rates in *An. gambiae* *s.l*. were lower in houses with livestock than those without livestock. This effect was significant when distances were incorporated in the analysis. The density of cattle may also be a potential risk factor for malaria. For instance, in Ethiopia, households with more cattle were associated with an increase in anopheline vector densities and HBI [15].

Therefore, the presence of cattle in rural areas could have a major impact on malaria transmission. One way cattle could impact malaria transmission is through zooprophylaxis, which is defined as “the use of wild or domestic animals, which are not the reservoir hosts of a given disease, to divert the blood-seeking mosquito vectors from the human hosts of that disease” [16]. By blood feeding on such animals, mosquitoes would have a reduced chance of acquiring malaria parasites. A number of studies have supported the hypothesis of zooprophylaxis [17–23] with some studies showing that the effect depends on cattle being corralled away from human dwellings [20, 22].

Alternatively, cattle could impact malaria transmission through zoopotentiation, i.e. the presence of cattle may create additional blood meal sources, and, as a result, the lifespan of the vectors can increase as well as their densities [18]. For instance, in Pakistan [6], The Gambia [14, 24], Ethiopia [25] and Lao PDR [26], the presence of cattle was associated with more malaria vectors and higher risk of malaria [5]. The question whether the presence of cattle reduces or enhances the risk of malaria transmission is likely dependent on the dominant malaria vector species, abundance of other potential hosts and the proximity of cattle to human hosts. For instance, a review by Donnelly et al*.* [18] found that zooprophylaxis would be effective in regions where the dominant vectors do not prefer to feed on human hosts and where livestock are kept at a distance away from humans at night. A model has also suggested that the presence of cattle near human dwellings would provide sufficient blood meals for the vectors, a phenomenon that would enhance the reproductive success of malaria vectors, thereby increasing the abundance of malaria vectors and the risk of malaria transmission [27]. Furthermore, the presence of cattle may increase the breeding habitats of malaria vectors as hoof prints of cattle may serve as additional suitable larval habitats leading to an increase in the density of vectors especially during the rainy season [27–29].

Therefore, more studies are needed to evaluate: (a) whether the presence of cattle would have an impact on the abundance and feeding behaviour of malaria vectors and (b) the distances at which livestock should be corralled to promote zooprophylaxis and prevent zoopotentiation. The present study aimed at assessing the effect of household-level cattle presence and distribution on the abundance of indoor and outdoor resting malaria vectors.

## Methods

### Study site

**Fig. 1:**
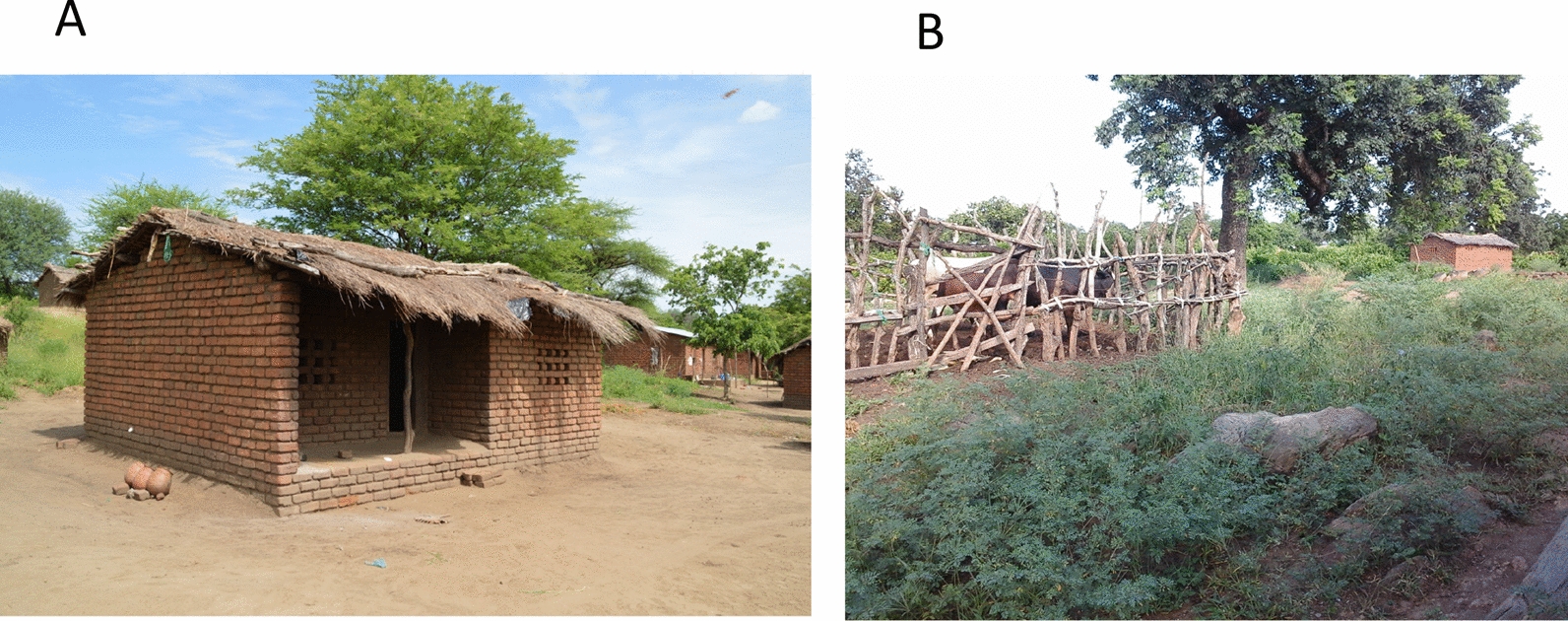
Typical house in the study region with **A** three clay pots set outdoors, close to each other and on the left side of the house and **B** cattle in a cattle-corral

The study was conducted in eight villages in Chikwawa district, southern Malawi, a low-lying region with high rates of malaria transmission [30, 31]. The region experiences a single rainy season from November through April, and the main malaria vectors prevalent in the region are *An. gambiae* *s.s*., *An. funestus* *s.s*. and *An. arabiensis* [31–33]. Malaria transmission occurs throughout the year with rates intensifying during the rainy season. The National Malaria Control Programme in Malawi conducted a mass distribution of ITNs in April 2016; about seven months prior to the start of the current study. Most of the houses are made of sun-dried or fire-baked bricks with grass thatched or corrugated iron-sheet roofs. Residents of this region engage in subsistence farming with maize and millet as the main crops. Many residents also keep domestic animals, including cattle, goats and pigs. Cattle marketing is an important economic activity for Chikwawa district, most likely due to the availability of water and feed for the cattle. About 21% of households in the study villages own cattle (range per village: 8–35%; Additional file 1: Table S1), with the main driver of cattle ownership likely being socioeconomic status. No single ethnic group is associated with livestock ownership in Chikwawa district. Common conditions for keeping cattle in the study villages included open/unroofed corrals as shown in Fig. 1B.

### Selection of households

The eight villages included in this study were part of a cluster-randomized trial assessing the effects of larval source management and house improvement on malaria transmission [34]. Household-level inclusion criteria were applied to allow for a certain level of uniformity across the houses. The criteria included: houses with open eaves, houses that were ≥ 25 m apart, and houses more than 100 m from any mosquito breeding habitat. From these eligible houses, houses without and with cattle corralled overnight within 50 m of the house, were selected. The first house at the start of the study was purposefully selected by a member of the research team. For the subsequent selections, the owner of each house would randomly select the next house to be sampled by selecting a piece of paper from an envelope that had 120 pieces of papers that had been folded and pre-labelled indicating ‘cattle’ or ‘no cattle’. Depending on the result (cattle or no cattle), the next nearest house that fit the criterion would be chosen.

### Mosquito sampling

Mosquito sampling was done from November 2016 through March 2017, which is the rainy season in Malawi. The sampling included indoor and outdoor resting collections in eight villages, in 100 houses, 40 of which had no cattle and 60 of which had cattle. Each house was sampled on two consecutive days, resulting in 200 house-nights of sampling. Out of the eight villages, the number of sampled houses in each village ranged from three to seven, which was based on the ability to cover all the houses before 10:00 hrs, a period when mosquitoes are still resting indoors. Clay pots [35] were used for the outdoor collections, whereby three pots were set outside close to each other on the left side of the house and 1 m away from the wall of the house, starting in the evening to the following morning (Fig. 1A). The mosquitoes resting in the clay pots were collected the following morning from 07:00 hrs to 10:00 hrs by covering the pot with a cotton cloth and dropping a cotton ball soaked with chloroform to anesthetize the mosquitoes. After 4–5 min, the mosquitoes were collected from the clay pots and placed in perforated 1.5 ml Eppendorf tubes that were then placed in containers with a desiccant. Prokopack aspirators [36] were used for the indoor collections. These collections were conducted from 07:00 hrs to 10:00 hrs, on the same morning as mosquitoes were collected from the clay pots, by an individual who actively searched for mosquitoes in all the rooms for a maximum of 10 min per house. Mosquitoes collected indoors were also stored in perforated 1.5 ml Eppendorf tubes that were then placed in containers with a desiccant. The containers were assigned a unique code to distinguish the indoor and outdoor collections, the day of collection and the specific house. In houses with cattle, the number of cattle and the distances from the house to where the cattle were corralled the previous night were recorded. Furthermore, brief interviews were conducted with householders to obtain data on additional factors that may have influenced the resting behaviour of mosquitoes. The factors were: the number of people that occupied the house the previous night, use of bed nets, wall type, floor type, door type and cooking locations. The following represent the categorizations: door type as wood or reed; floor type as dirt/mud/dung/sand, wood/plank, cement or tiles; wall type as sun-dried bricks or fire-baked bricks; cooking location as inside the house, on the veranda, outside but within 2 m of the house or outside more than 2 m away from the house.

### Mosquito identification

In the laboratory, all mosquitoes were identified morphologically using the key from Gillies and Coetzee [37]. All anophelines were classified as *An. gambiae* *s.l*., *An. funestus* *s.l*. or *Anopheles coustani.* There was no further classification of the culicines beyond subfamily level. Each identified mosquito was placed in 1.5 ml Eppendorf tube with a desiccant. Females from the *An. gambiae* *s.l*. species complex and *An. funestus* *s.l*. species group were further identified to species level using the polymerase chain reaction (PCR) method [38–40]. For the *An. gambiae* species complex, the PCR included species-specific primers for *An. gambiae* *s.s*., *An. arabiensis*, and *Anopheles quadriannulatus*. For the *An. funestus* species group, the PCR included species-specific primers for *An. funestus* *s.s*., *Anopheles vandeeni*, *Anopheles rivulorum*, *Anopheles rivulorum-*like, *Anopheles parensis*, and *Anopheles leesoni*. The heads and thoraces of all female *An. gambiae* *s.l*. and *An. funestus* *s.l*. were tested for the presence of *Plasmodium falciparum* DNA using real-time PCR [41], with a Ct value ≤ 37.0 as the cut-off for *P. falciparum* positive. The abdomens of all fed and half-gravid female *An. gambiae* *s.l*. and *An. funestus* *s.l*. were analysed using PCR to identify the blood meal host. The PCR included species-specific primers for human, cow, goat, pig and dog [42], as well as general primers designed for mammal and avian hosts [43] when species-specific primers did not amplify.

### Data analysis

Generalized linear models were fitted with a Poisson distribution to compare the mean catches of mosquitoes per night: (a) in houses with and without cattle present at the household-level, (b) across the average distances from the house to where the cattle were corralled the previous night and (c) on cattle densities at the household-level. Catches of female *An. gambiae* *s.l*., *An. funestus* *s.l*. and culicines were treated as dependent variables in separate fitted models. For some houses, cattle were corralled in more than one location near the house; therefore, average distances were calculated by summing the distances from the house to where the cattle were corralled the previous night and dividing by the total number of cattle. Average densities of cattle were also calculated. This was done by summing the total number of cattle within 50 m of a house and dividing by the total number of locations where those cattle were kept overnight. The cooking locations, number of people that slept in the house the previous night and the use of bed net were included as covariates in each of the models. Doors and sibling species were not included in the analysis because all the doors were made of wood and sibling species were too few to carry out meaningful analysis. Generalized estimating equations were used to account for the repeated measures by house in each of the models. The datasets were analysed using SPSS Version 20.0.

## Results

Combined across all locations, a total number of 571 mosquitoes was collected. Of these, 300 were males (anophelines: 13 indoors and 5 outdoors; culicines: 278 indoors and 4 outdoors) and 271 were females. Of the 271 females, 190 were culicines (179 indoors, 11 outdoors) and 81 were anophelines (63 fed, 13 half-gravid, 3 gravid and 2 unfed). Of the 81 anopheline females, 48 were *An. gambiae* *s.l*. (33 indoors and 15 outdoors), 32 were *An. funestus* *s.l*. (30 indoors and 2 outdoors) and 1 was *An. coustani* (1 outdoors; Table 1). Of the 63 anopheline females caught indoors, 60 were identified by PCR: *An. arabiensis* (n = 25), *An. gambiae* *s.s*. (n = 6) and *An. funestus* *s.s*. (n = 29). The DNA of the remaining three anophelines caught indoors failed to amplify (2 *An. gambiae* *s.l*.; 1 *An. funestus* *s.l*.). Of the 17 *An. gambiae* *s.l*. and *An. funestus* *s.l*. females caught outdoors, 13 were identified by PCR: *An. arabiensis* (n = 11), *An. rivulorum-*like (n = 1) and *An. funestus* *s.s*. (n = 1). The DNA of the remaining four anophelines caught outdoors failed to amplify (4 *An. gambiae* *s.l*.).

**Table 1:** Mosquito collections in houses with and without cattle

Mosquito resting collections					
Houses	With cattle		Without cattle		Total
No. of house-nights sampled	120		80		200
	Indoors	Outdoors	Indoors	Outdoors	
Female *An. arabiensis*	13	8	12	3	36
Female *An. gambiae* *s.s*.	3	0	3	0	6
Female *An. gambiae* *s.l*.^a^	1	2	1	2	6
Female *An. funestus* *s.s*.	11	1	18	0	30
Female *An. rivulorum*-like	0	1	0	0	1
Female *An. funestus* *s.l*.^a^	1	0	0	0	1
Female *An. coustani*	0	0	0	1	1
Female culicines	87	6	92	5	190
Male anophelines	6	2	7	3	18
Male culicines	174	3	104	1	282

^a^*An. gambiaes.l*. and *An. funestuss.l*.: specimens did not amplify with species-specific PCR and therefore could not be identified further

Of the 80 *An. gambiae* *s.l*. and *An. funestus* *s.l*. females tested for the presence of *P. falciparum* DNA, only one was positive for *P. falciparum* (*An. arabiensis*, indoor, fed on human blood)*.*

Of the 81 anopheline females, 75 (62 fed and 13 half -gravid) were tested to identify the blood meal hosts. Twenty-five blood meals were identified to the species level: cow (n = 22; 18 *An. arabiensis*, 1 *An. gambiae* *s.s*., 2 *An. gambiae* *s.l*. and 1 *An*. *funestus* *s.s*.); goat (n = 2; 1 *An. arabiensis*, 1 *An. rivulorum-*like) and human (n = 1; *An. arabiensis*). Seventeen blood meals amplified using the general mammal primer but did not amplify with the human, cow, goat, pig or dog primers (1 *An. gambiae* *s.s*., 5 *An. arabiensis*, 11 *An. funestus* *s.s*.). Thirty-three of the seventy-five blood meals failed to amplify (Table 2).

**Table 2:** Blood meal analysis results

Female anophelines	Cattle presence (a) or absence (b)	Cow		Human		Mammal		Goat		No amplifications	
		In	Out	In	Out	In	Out	In	Out	In	Out
*An. gambiaes.l*.	a	0	1	0	0	0	0	0	0	1	1
	b	0	1	0	0	0	0	0	0	1	1
*An. funestuss.l*.	a	0	0	0	0	0	0	0	0	1	0
	b	0	0	0	0	0	0	0	0	0	0
*An. arabiensis*	a	8	3	0	0	3	0	0	0	2	5
	b	6	1	1	0	1	1	1	0	2	1
*An. gambiaes.s*.	a	0	0	0	0	0	0	0	0	3	0
	b	1	0	0	0	1	0	0	0	0	0
*An. funestuss.s*.	a	0	0	0	0	2	1	0	0	6	0
	b	1	0	0	0	8	0	0	0	9	0
*An. rivulorum-*like	a	0	0	0	0	0	0	0	1	0	0
	b	0	0	0	0	0	0	0	0	0	0
Totals		22		1		17		2		33	

*An. gambiaes.l*. and *An. funestuss.l*.: species-specific PCR failed to amplify

Mammal: blood meals were positive for mammal-specific PCR, but did not amplify for human, cattle, goat, pig or dog

**Fig. 2:**
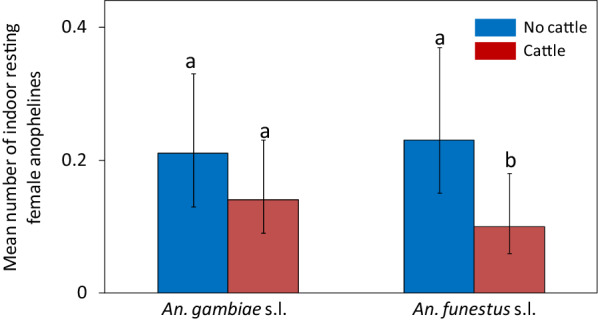
Effect of cattle presence or absence on the mean number of female anophelines caught resting indoors per house-night. Bars with different letters denote significant differences in the number of mosquitoes collected. Error bars are the 95% confidence interval

**Fig. 3:**
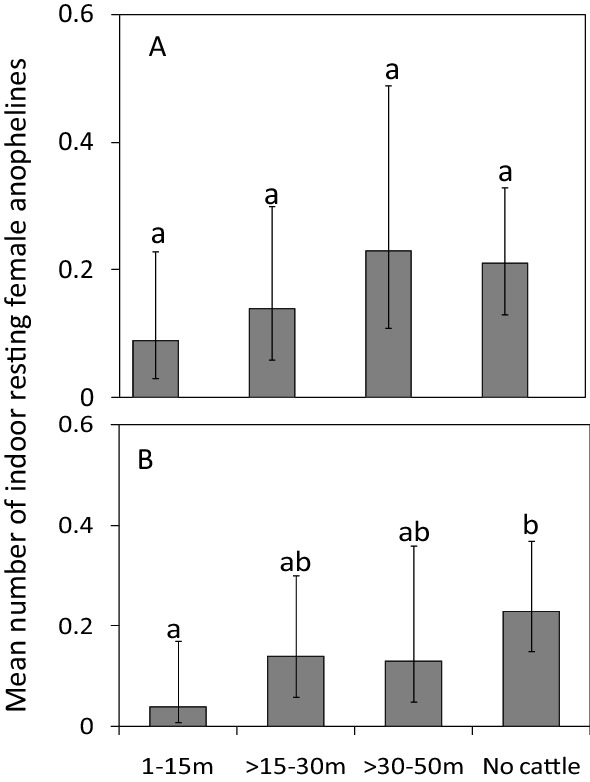
Effect of cattle presence at various distances, or absence, on the mean number of female **A** *An. gambiae* *s.l*. **B** *An. funestus* *s.l*. caught resting indoors per house-night. Bars with different letters denote significant differences in the number of mosquitoes collected. Error bars are the 95% confidence interval

The abundance of *An. gambiae* *s.l*. resting indoors was not different between houses with and without cattle [risk ratio (RR) = 0.70, 95% confidence interval (CI) = (0.34–1.45), P = 0.34]. The abundance of *An. funestus* *s.l*. resting indoors was lower in houses with cattle than in houses without cattle [RR = 0.46, CI = (0.21–1.0), P = 0.04] (Fig. 2). Compared to houses without cattle, the presence of cattle at various distances did not have an impact on catch sizes of *An. gambiae* *s.l*.: 1–15 m (RR = 0.41, CI = 0.13–1.26, P = 0.12); > 15–30 m (RR = 0.66, CI = 0.24–1.80, P = 0.42) and > 30–50 m (RR = 1.20, CI = 0.47–3.05, P = 0.70; Fig. 3A). However, compared to houses without cattle, there was a reduction in the catch sizes of indoor resting *An. funestus* *s.l*. when cattle were present at average distances of 1–15 m [RR = 0.19, CI = (0.04–0.86), P = 0.03]. As the average distances increased, the catch sizes of this species were similar to those of houses without cattle: average distances > 15–30 m (RR = 0.58, CI = 0.22–1.55, P = 0.28) and > 30–50 m (RR = 0.73, CI = 0.23–2.29, P = 0.59; Fig. 3B). The number of people that slept in the house the previous night, use of bed net and cooking locations did not have an effect on the abundance of malaria vectors in houses with and without cattle (Additional file 2: Table S2, Additional file 3: Table S3, Additional file 4: Table S4 and Additional file 5: Table S5).

**Fig. 4:**
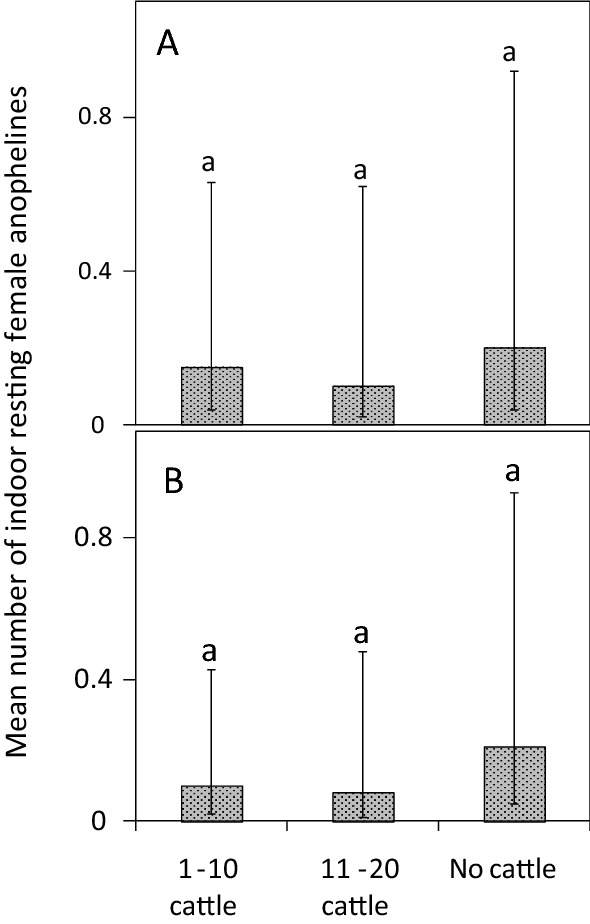
Effect of cattle density on the mean number of female **A** *An. gambiae s.l*. and **B** *An. funestus* *s.l*. caught resting indoors per house-night. Error bars are the 95% confidence interval

Houses without cattle and those with fewer cattle (average densities of 1–10) had similar catches of indoor resting *An. gambiae* *s.l*. [RR = 0.74, CI = (0.35–1.55), P = 0.42; Fig. 4A] and *An. funestus* *s.l*. [RR = 0.48, CI = (0.21–1.08), P = 0.08; Fig. 4B]. Additionally, houses with more cattle (average densities of 11–20) did not differ from houses without cattle in the abundance of indoor resting *An. gambiae* *s.l*. [RR = 0.52, CI = (0.12–2.37), P = 0.40] or *An. funestus* *s.l*. malaria vectors [RR = 0.39, CI = (0.09–1.75), P = 0.22; Fig. 4A, B, respectively]. As clay pots yielded low catch sizes of malaria vectors outdoors (n = 18), a statistical analysis was not possible.

**Fig. 5:**
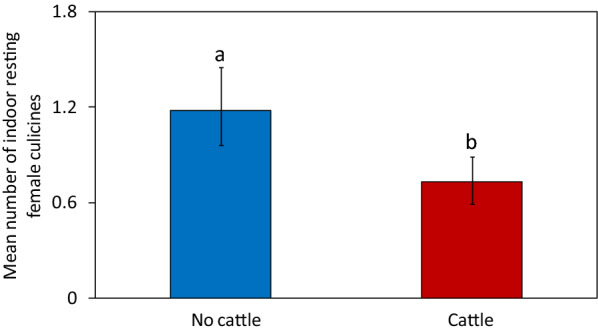
Effect of cattle presence or absence on the mean number of female culicines caught resting indoors per house-night. Bars with different letters denote significant differences in the number of mosquitoes collected. Error bars are the 95% confidence interval

**Fig. 6:**
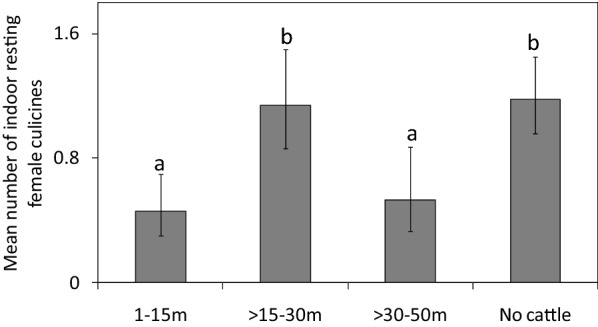
Effect of cattle presence at various distances, or absence, on the mean number of female culicines caught resting indoors per house-night. Bars with different letters denote significant differences in the number of mosquitoes collected. Error bars are the 95% confidence interval

For the indoor resting female culicines, the catch sizes of these mosquitoes were lower in houses with cattle than in houses without cattle (RR = 0.73, CI = 0.53–1.01, P = 0.001; Fig. 5). Additionally, compared to houses without cattle, the presence of cattle reduced the abundance of indoor resting culicines at an average distance 1–15 m [RR = 0.46, CI = (0.28–0.75), P = 0.002]; and 30.01–50 m [RR = 0.62, CI = (0.36–1.07), P = 0.003]. However, compared to houses without cattle, the catches of indoor resting culicines were similar to those caught near houses with cattle at an average distance of > 15–30 m [RR = 1.12, CI = (0.76–1.61), P = 0.60; Fig. 6]. Interestingly, the catch sizes of indoor resting female culicines (n = 179) were lower than those of the male culicines (n = 278) indoors (Table 1).

## Discussion

To our knowledge, this is the first study in southern Malawi to explore, at household-level, the impact of cattle on the resting behaviour of malaria vectors. The most abundant vectors caught resting indoors were *An. gambiae* *s.l.* (primarily *An. arabiensis*) and *An. funestus* *s.l*. (primarily *An. funestus* *s.s*.). For the outdoor resting collections, the most abundant vector was *An. arabiensis*. The presence of cattle was associated with a reduction in the abundance of *An. funestus* *s.l*. mosquitoes resting indoors, and this reduction was strongest when cattle were 1–15 m away from the house. The abundance of indoor resting *An. gambiae* *s.l*. (i.e. *An. arabiensis*) was not apparently affected by the presence of cattle, even after accounting for the distances from the house to where the cattle were corralled the previous nights. Similar to other studies [28], the density of cattle did not have an effect on the abundance of indoor and outdoor resting malaria vectors. These results demonstrate that the presence of cattle near a house influences the abundance of indoor resting malaria vectors such as *An. funestus* *s.s*., independent of cattle density. Of those blood meal hosts that could be identified, most of the *An. arabiensis* mosquitoes had fed on cattle blood. Only one *An. arabiensis* was shown to have fed on human blood. The host species of most *An. funestus* *s.s*. blood meals in this study could not be identified. Surprisingly, 11 *An. funestus* *s.s*. blood meals were positive for mammalian blood other than from a human, cattle, goat, dog or pig. Further studies are needed to thoroughly understand the host-feeding preference of malaria vectors in this region.

*Anopheles funestuss.s*. is known to be highly anthropophagic [44–54], so the finding that the density of this species was reduced in houses near cattle was unexpected. One potential reason could be that cattle odour had a deterrent or a masking effect on this species. A high degree of aversion to cattle odour has been reported for *An. gambiae* *s.s*., which is also anthropophagic [55]. Therefore, it is possible that when cattle are close to a house in this region of southern Malawi, their odours cause aversion of *An. funestus* *s.s*. from these houses. The reduction of *An. funestus* *s.s*. in houses near cattle has a potential to reduce malaria transmission. However, studies incorporating the vectors’ host-seeking behaviour/human biting rates are recommended to fully support this finding. Additionally, the finding supports the fact that, exploration of odours that have a repellent effect on malaria vectors helps in developing synthetic repellents for use when people are not protected by LLINs. For instance, in India, the use of cow dung-based mosquito repellent has been developed [56, 57]. On the other hand, *An. arabiensis* is an opportunistic feeder, typically feeding on cattle or humans indiscriminately [8]. For instance, in Ethiopia, this species has been found to feed on cattle outdoors but still uses the house as a resting site [17]. It is, therefore, not surprising that the abundance of this species resting indoors was not affected by the presence of cattle because cattle are suitable hosts. However, this finding warrants further studies because in the current study, the abundance of mosquitoes was very low, which may have limited the ability to detect a difference. For future studies, combining resting collections with host-seeking behavioural studies is highly recommended. While data on mosquito resting abundance and host choice are useful to infer how mosquitoes respond to the presence of alternative hosts, assessment of the epidemiological impact will require direct measurement of biting rates on humans, which is best estimated by host-seeking collections to assess entomological inoculation rates.

The study was carried out in traditional houses spread across eight villages allowing for comparisons of the resting behaviour of mosquitoes in houses with and without cattle under natural conditions. While inclusion criteria were used to reduce variation and increase comparability among the houses, additional factors that could not be controlled, but which may have influenced the resting behaviour of mosquitoes, were included as covariates in the statistical analyses. None of these covariates had an effect on the abundance of indoor-resting malaria vectors across the eight villages.

Identification of blood meals in this study found only one *An. arabiensis* that had fed on humans, with a larger number of the mosquitoes that amplified being positive for cattle blood. However, the DNA from quite a number of *An. arabiensis* failed to amplify. Therefore, these results should be interpreted with caution. *Anopheles funestus* *s.s*. mosquitoes found resting indoors were mainly positive with mammalian blood which could not be identified further. This finding suggests that these mosquitoes may have fed outdoors but still used a house as a resting site, an example of exophagic–endophillic behaviour that is not typically associated with this species. Previous reports from Tanzania also observed exophagic–endophillic behaviour in *An. funestus* *s.s*. and *An. rivulorum* that had fed on goat blood [58].

The study had some limitations. Overall, the abundance of malaria vectors in this region was low, which reduced the power of some analyses and limited the generalizability of the results to other settings. Secondly, half of the blood meals failed to amplify for identification of the blood source. Some studies conducted in Tanzania [58] and Zambia [59] have experienced similar challenges although fewer samples failed to amplify in these studies than in the current study. Potential reasons that blood meals could not be identified include: (i) mosquitoes fed on species other than those included in our primer set, (ii) mosquitoes had incomplete blood meals or (iii) the DNA of the blood meal host was degraded. In this study, mosquitoes were collected early in the morning, at a time when blood meals are still relatively “fresh”, and the mosquitoes were immediately placed with a desiccant to preserve the DNA of blood meal hosts. Despite this effort, degradation of DNA of the blood meal host may have occurred. Furthermore, the specific mammal could not be identified further, most likely because the specific host could not be detected by the available primers in the current study.

The findings demonstrate that in the current study area, differences exist between malaria vector species and their interactions with potential blood meal hosts. This may have implications for malaria risk because whereas *An. funestus* *s.s*. was less often found resting indoors when cattle were in close proximity to a house, *An. arabiensis* remained unaffected. The reduction of *An. funestus* *s.s*. resting in houses with cattle nearby suggests a potential protective effect of cattle, but requires additional studies. Furthermore, as quite a number of the *An. funestus* *s.s*. population fed on mammals other than humans, cattle, goats or pigs, this may indicate that *An. funestus* *s.s*. in this region exhibits a zoophagic–anthropophagic trait similar to a previous finding in Madagascar [60]. Overtime, due to insecticide pressure as a consequence of people being protected by LLINs, a fraction of *An. funestus* *s.s*. in the study region may become zoophagic, as seen in Madagascar and western Kenya [48, 60].

The catch sizes of indoor resting female culicines were lower in houses with cattle than in houses without cattle. It is possible that the presence of cattle near a house would prevent nuisance biting by the culicines, but the distances from a house to where the cattle are corralled need to be taken into consideration.

The outdoor collections with clay pots yielded lower catches of resting malaria vectors, which were primarily *An. arabiensis*. The low catches with clay pots are contrary to findings elsewhere [35, 61, 62], but similar to studies by Mmbando et al*.* [63], where resting buckets placed outdoors yielded relatively few *An. arabiensis*. Outdoor collections may be more prone to predation than indoor collections [64], but in the present study, the clay pots were dusted every day to remove any webs or insects. Therefore, predation of mosquitoes from the clay pots was unlikely. Most likely, the mosquitoes sought to rest in alternative sites outdoors. This raises the need for the development of tools that can be effective in collecting outdoor resting mosquitoes such as resting buckets, boxes or sticky boxes [4, 65–67]. Additionally, tools that can target different outdoor sites are highly recommended as recent studies have shown that mosquitoes mostly prefer to rest inshady sites [62].

## Conclusion

In southern Malawi, differences in the abundance and resting habits of *An. arabiensis* and *An. funestus* *s.s*. were found. This may have implications for malaria risk because, whereas *An. funestus* *s.s*. was less often found resting indoors when cattle were in close proximity to a house, *An. arabiensis* remained unaffected. The reduction of *An. funestus* *s.s*. in houses near cattle has the potential to reduce malaria transmission. However, the low abundance of mosquitoes reduced the power of some analyses and limited the generalizability of the results to other settings. Therefore, studies incorporating the vectors’ host-seeking behaviour/human biting rates are recommended to fully support this finding.

## Supplementary Information

**Additional file 1: Table S1.** Cattle ownership in the eight villages.

**Additional file 2: Table S2.** Effect of cattle presence or absence on indoor resting female mosquitoes.

**Additional file 3: Table S3.** Effect of cattle presence at various distances, or absence, on indoor resting mosquitoes.

**Additional file 4: Table S4.** Effect of cattle density on indoor resting female anophelines.

**Additional file 5: Table S5.** Characteristics of houses with and without cattle.

## Data Availability

The datasets for this study are available upon a reasonable request.

## Abbreviations

LLINs: Long-lasting insecticidal nets
HBI: Human Blood Index
PCR: Polymerase chain reaction
RR: Risk ratio
CI: Confidence interval
WHO: World Health Organization
