# Supplementary material for: Impact of cattle on the abundance of indoor and outdoor resting malaria vectors in southern Malawi
Source: Malar J. 2021 Aug 26;20:353. doi: 10.1186/s12936-021-03885-x (PMC8390081; doi:10.1186/s12936-021-03885-x)
Supplement: Supplementary file 1 — Additional file 1: Table S1. Cattle ownership in the eight villages. [file 12936_2021_3885_MOESM1_ESM.docx]

Table S1: Cattle ownership in the eight study villages.

| Village | Households with cattle | (%) | Households without cattle | (% ) | Total households surveyed |
| --- | --- | --- | --- | --- | --- |
| BWA | 23 | (29%) | 56 | (71%) | 79 |
| C4H | 12 | (14%) | 71 | (86%) | 83 |
| K4I | 10 | (12%) | 73 | (88%) | 83 |
| KAI | 38 | (35%) | 71 | (65%) | 109 |
| LIW | 7 | (8%) | 81 | (92%) | 88 |
| MAI | 43 | (30%) | 101 | (70%) | 144 |
| MWA | 20 | (21%) | 74 | (79%) | 94 |
| SEK | 8 | (11%) | 63 | (89%) | 71 |
| Total | 161 | (21%) | 590 | (79%) | 751 |

Households were randomly selected for participation in a rolling malaria indicator survey (rMIS) between April 2015 and May 2018 as described in McCann *et al.* (2021). As part of the survey, householders were asked about cattle ownership.Only villages from the current study are included here. For households participating in multiple rounds of the rMIS, only the first record is included here.
