# Supplementary material for: Impact of cattle on the abundance of indoor and outdoor resting malaria vectors in southern Malawi
Source: Malar J. 2021 Aug 26;20:353. doi: 10.1186/s12936-021-03885-x (PMC8390081; doi:10.1186/s12936-021-03885-x)
Supplement: Supplementary file 2 — Additional file 2: Table S2. Effect of cattle presence or absence on indoor resting female mosquitoes. [file 12936_2021_3885_MOESM2_ESM.docx]

|  | *An. gambiae* s.l. | | | *An. funestus* s.l. | | | Female culicines | | |
| --- | --- | --- | --- | --- | --- | --- | --- | --- | --- |
| **Treatments** | **P-value** | **RR** | **95% CI** | **P-value** | **RR** | **95% CI** | **P-value** | **RR** | **95% CI** |
| Cattle presence | 0.34 | 0.70 | 0.34 – 1.45 | 0.04 | 0.46 | 0.21 – 1.0 | 0.01 | 0.73 | 0.53 – 1.01 |
| Cattle absence* |  |  |  |  |  |  |  |  |  |
| People that slept in the house the previous night | 0.71 | 0.96 | 0.76 – 1.20 | 0.40 | 1.11 | 0.88– 1.39 | 0.37 | 1.04 | 0.95 – 1.15 |
| Mosquito control_bednet | 0.86 | 0.92 | 0.39 – 2.21 | 0.42 | 1.56 | 0.53 – 4.62 | 0.00 | 0.49 | 0.35– 0.67 |
| Mosquito control_none* |  |  |  |  |  |  |  |  |  |
| Cooking inside the house | 0.74 | 0.84 | 0.30 – 2.36 | 0.15 | 2.19 | 0.76 – 6.32 | 0.00 | 2.5 | 1.66 – 3.80 |
| Cooking on the veranda | 0.56 | 0.75 | 0.28 – 1.99 | 0.69 | 1.25 | 0.42 -3.75 | 0.57 | 1.15 | 0.72 – 1.82 |
| Cooking outside, within 2m of the house | 0.97 | 0.99 | 0.40 – 2.40 | 0.57 | 1.36 | 0.47 – 3.97 | 0.49 | 1.18 | 0.75 – 1.85 |
| Cooking outside, away from 2m of the house * |  |  |  |  |  |  |  |  |  |
| - denotes the reference | | | | | | | | | |

Table S2: Effect of cattle presence or absence on indoor resting female mosquitoes
