# Supplementary material for: Impact of cattle on the abundance of indoor and outdoor resting malaria vectors in southern Malawi
Source: Malar J. 2021 Aug 26;20:353. doi: 10.1186/s12936-021-03885-x (PMC8390081; doi:10.1186/s12936-021-03885-x)
Supplement: Supplementary file 3 — Additional file 3: Table S3. Effect of cattle presence at various distances, or absence, on indoor resting mosquitoes. [file 12936_2021_3885_MOESM3_ESM.docx]

|  | *An. gambiae* s.l. | | | *An. funestus* s.l. | | | Female culicines | | |
| --- | --- | --- | --- | --- | --- | --- | --- | --- | --- |
| **Distances** | **P-value** | **RR** | **95% CI** | **P-value** | **RR** | **95% CI** | **P-value** | **RR** | **95% CI** |
| 1-15m | 0.12 | 0.41 | 0.13-1.26 | 0.03 | 0.19 | 0.04-0.86 | 0.002 | 0.46 | 0.28-0.75 |
| ≥15-30m | 0.42 | 0.66 | 0.24-1.80 | 0.28 | 0.58 | 0.22-1.55 | 0.60 | 1.12 | 0.76-1.61 |
| ≥30-50m | 0.70 | 1.20 | 0.47-3.05 | 0.59 | 0.73 | 0.23-2.29 | 0.03 | 0.62 | 0.36-1.07 |
| No cattle* |  |  |  |  |  |  |  |  |  |
| People that slept in the house the previous night | 0. 95 | 1.01 | 0.80-1.27 | 0. 32 | 1.13 | 0.89-1.43 | 0. 63 | 1.03 | 0.93-1.13 |
| Mosquito control_bednet | 0.73 | 0.86 | 0.35-2.07 | 0. 43 | 1.55 | 0.52-4.60 | 0. 00 | 0.50 | 0.36-0.70 |
| Mosquito control_none* |  |  |  |  |  |  |  |  |  |
| Cooking inside the house | 0.84 | 0.90 | 0.31-2.56 | 0. 15 | 2.22 | 0.76-6.49 | 0. 00 | 2.30 | 1.52-3.49 |
| Cooking on the veranda | 0.50 | 0.71 | 0.27-1.90 | 0. 83 | 1.13 | 0.37-3.40 | 0. 86 | 1.04 | 0.65-1.67 |
| Cooking outside, within 2m of the house | 0.90 | 1.06 | 0.43-2.63 | 0. 57 | 1.37 | 0.46-4.04 | 0. 82 | 1.06 | 0.67-1.67 |
| Cooking outside, away from 2m of the house * |  |  |  | ^-^ |  |  | ^-^ |  |  |
| - denotes the reference | | | | | | | | | |

Table S3: Effect of cattle presence at various distances, or absence, on indoor resting mosquitoes
