# Supplementary material for: Impact of cattle on the abundance of indoor and outdoor resting malaria vectors in southern Malawi
Source: Malar J. 2021 Aug 26;20:353. doi: 10.1186/s12936-021-03885-x (PMC8390081; doi:10.1186/s12936-021-03885-x)
Supplement: Supplementary file 4 — Additional file 4: Table S4. Effect of cattle density on indoor resting female anophelines. [file 12936_2021_3885_MOESM4_ESM.docx]

|  | *An. gambiae* s.l. | | | *An. funestus* s.l. | | |
| --- | --- | --- | --- | --- | --- | --- |
| **Cattle density** | **P-value** | **RR** | **95% CI** | **P-value** | **RR** | **95% CI** |
| 1-10 cattle | 0.42 | 0.74 | 0.35-1.55 | 0.08 | 0.48 | 0.21-1.08 |
| 11-20 cattle | 0.39 | 0.52 | 0.12-2.37 | 0.22 | 0.39 | 0.09-1.75 |
| No cattle* |  |  |  |  |  |  |
| People that slept in the house the previous night | 0.77 | 0.97 | 0.77-1.21 | 0.38 | 1.11 | 0.88-1.40 |
| Mosquito control_bednet | 0.84 | 0.92 | 0.38-2.20 | 0.43 | 1.55 | 0.52-4.61 |
| Mosquito control_none* |  |  |  |  |  |  |
| Cooking inside the house | 0.78 | 0.86 | 0.30-2.46 | 0.14 | 2.23 | 0.76-6.53 |
| Cooking on the veranda | 0.58 | 0.76 | 0.29-2.02 | 0.68 | 1.27 | 0.42-3.81 |
| Cooking outside, within 2m of the house | 0.99 | 1.0 | 0.41-2.43 | 0.56 | 1.37 | 0.47- 4.0 |
| Cooking outside, away from 2m of the house * |  |  |  | ^-^ |  |  |
| *denotes the reference | | | | | | |

Table S4: Effect of cattle density on indoor resting female anophelines
