# Supplementary material for: Impact of cattle on the abundance of indoor and outdoor resting malaria vectors in southern Malawi
Source: Malar J. 2021 Aug 26;20:353. doi: 10.1186/s12936-021-03885-x (PMC8390081; doi:10.1186/s12936-021-03885-x)
Supplement: Supplementary file 5 — Additional file 5: Table S5. Characteristics of houses with and without cattle. [file 12936_2021_3885_MOESM5_ESM.docx]

|  | **Houses with cattle (60)*** | **Houses without cattle (40)*** |
| --- | --- | --- |
|  |  |  |
| Mean number of people that slept in the house the previous night | 4 (65%) | 3 (35%) |
| Proportion of houses using mosquito-control_bednet | 81% | 81% |
| Proportion of houses not using mosquito-control_bednet | 19% | 19% |
| Proportion of houses cooking inside the house | 9% | 29% |
| Proportion of houses cooking on the veranda | 31% | 21% |
| Proportion of houses cooking outside, within 2m of the house | 33% | 22% |
| Proportion of houses cooking outside, further than 2m of the house | 27% | 28% |
| *each house was sampled on two nights | | |

Table S5: Characteristics of houses with and without cattle
